# Effects of dexamethasone on the Li-pilocarpine model of epilepsy: protection against hippocampal inflammation and astrogliosis

**DOI:** 10.1186/s12974-018-1109-5

**Published:** 2018-03-05

**Authors:** Adriana Fernanda K. Vizuete, Fernanda Hansen, Elisa Negri, Marina Concli Leite, Diogo Losch de Oliveira, Carlos-Alberto Gonçalves

**Affiliations:** Department of Biochemistry, Instituto de Ciências Básicas da Saúde, Universidade Federal do Rio Grande do Sul, Ramiro Barcelos, 2600-Anexo, Porto Alegre, RS 90035-003 Brazil

**Keywords:** Epilepsy, Dexamethasone, Neuroinflammation, Astrocytes, S100B

## Abstract

**Background:**

Temporal lobe epilepsy (TLE) is the most common form of partial epilepsy and is accompanied, in one third of cases, by resistance to antiepileptic drugs (AED). Most AED target neuronal activity modulated by ionic channels, and the steroid sensitivity of these channels has supported the use of corticosteroids as adjunctives to AED. Assuming the importance of astrocytes in neuronal activity, we investigated inflammatory and astroglial markers in the hippocampus, a key structure affected in TLE and in the Li-pilocarpine model of epilepsy.

**Methods:**

Initially, hippocampal slices were obtained from sham rats and rats subjected to the Li-pilocarpine model of epilepsy, at 1, 14, and 56 days after *status epilepticus* (SE), which correspond to the acute, silent, and chronic phases. Dexamethasone was added to the incubation medium to evaluate the secretion of S100B, an astrocyte-derived protein widely used as a marker of brain injury. In the second set of experiments, we evaluated the in vivo effect of dexamethasone, administrated at 2 days after SE, on hippocampal inflammatory (COX-1/2, PGE2, and cytokines) and astroglial parameters: GFAP, S100B, glutamine synthetase (GS) and water (AQP-4), and K^+^ (Kir 4.1) channels.

**Results:**

Basal S100B secretion and S100B secretion in high-K^+^ medium did not differ at 1, 14, and 56 days for the hippocampal slices from epileptic rats, in contrast to sham animal slices, where high-K^+^ medium decreased S100B secretion. Dexamethasone addition to the incubation medium per se induced a decrease in S100B secretion in sham and epileptic rats (1 and 56 days after SE induction). Following in vivo dexamethasone administration, inflammatory improvements were observed, astrogliosis was prevented (based on GFAP and S100B content), and astroglial dysfunction was partially abrogated (based on Kir 4.1 protein and GSH content). The GS decrease was not prevented by dexamethasone, and AQP-4 was not altered in this epileptic model.

**Conclusions:**

Changes in astroglial parameters emphasize the importance of these cells for understanding alterations and mechanisms of epileptic disorders in this model. In vivo dexamethasone administration prevented most of the parameters analyzed, reinforcing the importance of anti-inflammatory steroid therapy in the Li-pilocarpine model and possibly in other epileptic conditions in which neuroinflammation is present.

**Electronic supplementary material:**

The online version of this article (10.1186/s12974-018-1109-5) contains supplementary material, which is available to authorized users.

## Background

Epilepsy is a neuronal disorder characterized by recurrent and spontaneous seizures, resulting from excessive, abnormal, and hypersynchronous neuronal activity [1–3]. Approximately 50 million people worldwide suffer from this neuronal disorder, which normally affect mostly children and the elderly population [4]. Temporal lobe epilepsy (TLE) that affects the limbic system [5, 6] is the most common form of partial epilepsy and resistance to anticonvulsive drugs which develops in about 30% of cases [7, 8]. The Li-pilocarpine-induced model of epilepsy exhibits similar hippocampal alterations to those observed in TLE patients [9, 10] and is accompanied by drug resistance [11].

Brain tissue samples from patients and experimental models show specific astroglial changes, mainly in the levels of glial fibrillary acidic protein (GFAP) and S100B protein [12–16]. In fact, results from several studies suggest that epileptogenesis involves changes in the glial cells beyond neuronal alterations [17, 18]. Astrocytes are glial cells that interact with neurons and form tripartite synapses [19]. Some studies have strongly implicated astrocytes in the development of epileptic disorders [20–23]. Astrogliosis and neuroinflammation have been correlated to epileptogenesis, recurrent, and spontaneous seizures [24–29]. For this reason, specific astroglial targets (e.g., S100B, glutamine synthetase (GS), potassium channel Kir 4.1, and water channel AQP-4) have been investigated with a view to improving therapeutic approaches and the development of antiepileptic drugs [30].

The S100B protein is a glial protein that is a widely used marker for brain injury conditions, including epileptic disorders [31, 32], and indeed displays an augmented secretion during brain injury condition, working as a neurotrophic cytokine or simply as a damage-associated molecular pattern (DAMP) [33]. Moreover, S100B secretion is modulated by LPS [34] and anti-inflammatory drugs [35, 36]. Chronically elevated extracellular levels of S100B potentially lead to neurodegenerative processes [37, 38].

Therapy with corticosteroids, such as dexamethasone palmitate, has been used to treat refractory epilepsy in children [39]. Dexamethasone has been shown to reduce seizures in epileptic encephalopathy patients [40, 41]; while dexamethasone use has been evaluated in the Li-pilocarpine model of epilepsy [42, 43], its effect on astroglial targets has not been fully investigated. We hypothesized that dexamethasone mediates downregulation of S100B secretion and that this change could contribute to decrease neuroinflammation and astrogliosis during epileptogenesis. In fact, extracellular levels of S100B are elevated in Li-pilocarpine model of epilepsy [14].

Herein, we investigated the effect of dexamethasone on inflammatory and astroglial parameter in the Li-pilocarpine model of epilepsy. Firstly, we evaluated the modulation of S100B secretion by dexamethasone in acute hippocampal slices at 1, 14, and 56 days after the induction of status epilepticus (SE) by Li-pilocarpine administration in young rats. In this experimental model, these times correspond approximately to the acute, silent, and chronic phases, respectively [44]. In the second set of experiments, we administered intraperitoneal dexamethasone 1 day after SE and analyzed inflammatory and hippocampal astroglial parameters (S100B, GFAP, GS, GSH, AQP-4, and Kir 4.1) at 1 and 56 days afterward. Cerebrospinal fluid (CSF) and serum S100B were also determined.

## Methods

### Animals

Male *Wistar* rats, at postnatal day 27, were used in this study. We focused this study on young (27-day-old) rats to characterize glial changes from an age corresponding to childhood in rats [45]. It is important to mention that, at this age, rats have developed and matured their blood-brain barrier [46], energetic metabolism [47], and GABAergic neurotransmission [48]. Animals were obtained from our breeding colony (Department of Biochemistry, UFRGS) and maintained under controlled light and environmental conditions (12 h light/12 h dark cycle at a constant temperature of 22 ± 1 °C). Procedures were in accordance with the National Institutes of Health Guide for the Care and Use of Laboratory Animals (NIH Publications No. 80-23) following the regulations of the local animal house authorities and Committee of Animal Use of UFRGS (project number 24472).

This study was divided into two parts. The first was to analyze the S100B secretion in the acute hippocampal slices in animals submitted to the Li-pilocarpine epileptic model. The second part was to observe the in vivo effects of dexamethasone on inflammatory and astroglial parameters in the model (Additional file 1).

### Epilepsy model

Animals were subjected to the LiCl-pilocarpine model of TLE, according to Cavalheiro [9]. Briefly, the rats were treated intraperitoneally with lithium (LiCl, 3 mEq/kg, i.p.) 12–18 h prior to the administration of pilocarpine (45 mg/kg, i.p.) (Sigma, St. Louis, MO, USA). Sham animals also received LiCl at 12–18 h prior to saline (0.9% NaCl, i.p.) administration. Animals were monitored and classified into five stages of an epileptic seizure, according to Racine’s scale: (1) mouth and facial movement, (2) head nodding, (3) forelimb clonus, (4) rearing with forelimb clonus, and (5) rearing and falling with forelimb clonus.

We considered SE when animals reached stage 4 and stayed at this stage for more than 30 min. SE induction was stopped after 90 min by administration of diazepam (10 mg/kg, i.p.) followed by four administrations of HBSS medium (at 1.5, 7, 12, and 24 h after SE onset) containing (in mM) 137 NaCl, 0.63 Na_2_HPO_4_, 4.17 NaHCO_3_, 5.36 KCl, 0.044 KH_2_PO_4_, 1.26 CaCl_2_.2H_2_O, 0.041 MgSO_4_.7H_2_O, 0.049 MgCl_2_.6H_2_O, and 5.55 glucose, in order to promote a better animal recovery.

For further experiments, only animals that reached stage 4 and presented recurrent seizures were used. These animals were analyzed at different times—1, 14, and 56 days after pilocarpine injection—which correspond to the acute, latent, and chronic phases of epilepsy induced by the Li-pilocarpine model [49].

### First study—evaluation of S100B secretion in the acute hippocampal slices of SE rats

Sixty male rats were divided into (1) sham and (2) SE animals at different times (1, 14, and 56 days) after pilocarpine injection. These animals were killed by decapitation, and their brains were removed and placed in a cold saline medium of the following composition (in mM): 120 NaCl, 2 KCl, 1 CaCl_2_, 1 MgSO_4_, 25 HEPES, 1 KH_2_PO_4_, and 10 glucose, adjusted to pH 7.4. The hippocampi were dissected, and transverse slices of 0.3 mm were obtained using a McIlwain Tissue Chopper. Slices were then transferred immediately into 24-well culture plates, each well containing 0.3 ml of saline medium and only one slice. The medium was replaced every 15 min with fresh saline medium at room temperature. Following a 120-min equilibration period, the medium was removed and replaced with basal or specific treatments (high potassium—30 mM KCl; 0.1 μM dexamethasone; vehicle—0.01% DMSO) for 60 min at 30 °C on a warming plate [50].

### S100B secretion

S100B content in the supernatant was measured by ELISA, as described previously [51]. Briefly, 50 μl of sample plus 50 μl of Tris buffer were incubated for 2 h on a microtiter plate that was previously coated with monoclonal anti-S100B SH-B1 (Sigma-Aldrich, St. Louis, MO, USA). Polyclonal anti-S100 (Dako, Carpinteria, CA, USA) was incubated for 30 min, and peroxidase-conjugated anti-rabbit antibody was then added for a further 30 min. The color reaction with OPD was measured at 492 nm. The standard S100B curve ranged from 0.02 to10 ng/ml and data expressed as nanograms per milligram or nanograms per milliliter. Results are shown as percentages of the control.

### Lactate dehydrogenase assay

Slice integrity was evaluated by lactate dehydrogenase (LDH) kinetic activity using a commercial kit (BioClin, Brazil). The assay was performed according to the manufacturer’s instructions.

### Second study—effect of dexamethasone on the SE model

Sixty male rats received an administration of vehicle (DMSO, 0.1%, i.p.) or dexamethasone (10 mg/kg, i.p.) 24 and 36 h after SE induction by LiCl-pilocarpine administration. Groups of animals were divided into (1) sham + vehicle, (2) SE + vehicle, and (3) SE + dexamethasone at 1 and 56 days after pilocarpine injection. Herein, the animals were monitored between 1 and 56 days after SE induction and dexamethasone administration. They were video monitored every other day, 4–5 h for behavioral evaluation of occurrence of recurrent spontaneous seizures. All SE animals developed epileptic behavior, i.e., all animals exhibited, at least, score 2 in the Racine’s scale between 7 and 25 days after SE induction. Dexamethasone did not change epileptic behavior (see Additional file 2).

### Brain tissue, serum, and CSF samples

Rats were anesthetized by intraperitoneal injection of ketamine (75 mg/kg) and xylazine (10 mg/kg), and the blood was collected by cardiac puncture; serum was obtained by centrifuging at 1000×*g* for 10 min (Eppendorf 5402, Hamburg, Germany) before storing at − 80 °C. For ventricular access, the anesthetized rats were placed in a stereotaxic apparatus and cerebrospinal fluid (CSF) was obtained carefully by puncturing the cisterna magna with an insulin syringe. A maximum volume of 30 μl was collected over a 3-min period to minimize the risk of brain stem damage. The hippocampi were dissected, and transverse slices of 0.3 mm were obtained using a McIlwain Tissue Chopper as described above. Samples were stored at − 80 °C until biochemical and immunological assays.

### Cytokines and prostaglandin E2 measurement

The hippocampal slices were homogenized in phosphate buffer saline (PBS) containing (in mM) 50 NaCl, 18 Na_2_HPO_4_, and 83 NaH_2_PO_4_.H_2_O, pH 7.4, with 1 mM EGTA and 1 mM phenylmethylsulphonyl fluoride (PMSF), followed by centrifugation at 1000×*g* for 5 min at 4 °C. Cytokines were measured in supernatants using rat TNF-α, IL-1β, IL-10 (eBioscience, San Diego, USA), and PGE2 (Enzo Life Science, Farmingdale, NY, USA) ELISA kits. Serum TNF-α content was also evaluated. Data are expressed in picograms per milligram protein (tissue samples) or picogram per milliliter (serum).

### S100B measurement

Slices were homogenized in PBS with 1 mM EGTA and 1 mM PMSF. The S100B content in the CSF, serum, and brain tissue was measured by ELISA, as described above. Data are expressed in nanograms per milligram protein (tissue samples) or nanogram per milliliter (CSF and serum).

### GFAP measurement

GFAP content was measured by ELISA, as described previously [52]. The ELISA for GFAP was carried out by coating wells of 96-well plates with 100-μl samples containing 70 μg of protein overnight at 4 °C. Wells were incubated with a polyclonal anti-GFAP antibody (Dako, Carpinteria, CA, USA) from rabbit for 2 h, followed by incubation with a secondary antibody conjugated with peroxidase for 1 h, at room temperature. The color reaction with OPD was measured at 492 nm. The standard GFAP (Calbiochem, San Diego, CA, USA) curve ranged from 0.1 to 10 ng/ml. Data are expressed in nanogram per milligram protein.

### Glutamine synthetase activity

The enzymatic assay for glutamine synthetase (GS) was performed, as described previously [53] with modifications. Briefly, the hippocampal slices were homogenized in 50 mM imidazole buffer. Homogenates were then incubated with (mM) 50 imidazole, 50 hydroxylamine, 100 L-glutamine, 25 sodium arsenate dibasic heptahydrate, 0.2 ADP, and 2 manganese chloride, pH 6.2 for 15 min at 37 °C. The reactions were terminated by the addition of 0.2 ml of 0.37 M FeCl_3_, 200 mM trichloroacetic acid, and 670 mM HCl. After centrifugation, supernatant absorbance was measured at 530 nm. The standard γ-glutamylhydroxamate acid (Sigma-Aldrich, St. Louis, MO, USA) curve ranged from 0.1 to 10 mmol/ml. GS activity is expressed as μmol/h/mg protein.

### Glutathione content

Reduced glutathione (GSH) content was determined based on [50]. Briefly, slices were homogenized in sodium phosphate buffer (0.1 M, pH 8.0), and protein was precipitated with 1.7% meta-phosphoric acid. *O*-phthaldialdehyde (1 mg/ml methanol) (Sigma) was added to the supernatant at room temperature for 15 min. Fluorescence was measured using excitation and emission wavelengths of 350 and 420 nm, respectively. The standard calibration glutathione (Sigma-Aldrich, St. Louis, MO, USA) solution curve ranged from 0 to 500 μM. Glutathione results are expressed as nanomoles per milligram protein.

### Western blotting

Nitrocellulose membranes were blocked overnight at 4 °C with 2% bovine serum albumin (BSA) in Tris-buffered saline (TBS; in mM 10 Tris, 150 NaCl, pH 7.5, and 0.05% Tween 20®) and then incubated overnight at 4 °C in blocking solution containing the following antibodies: anti-Kir 4.1, anti-AQP-4, anti-COX1, anti-COX2 (diluted 1:1000), and anti-GS (diluted 1:10,000) (Santa Cruz Biotechnology, Inc., Dallas, TX, USA), and anti-actin (1:2000) (Sigma). Subsequently, the membranes were incubated for 1 h at room temperature in a solution containing horseradish peroxidase (HRP)-conjugated anti-rabbit IgG (diluted 1:10,000), HRP anti-mouse IgG (diluted 1:10,000) (GEHealthcare, Sao Paulo, Brazil), or HRP anti-goat diluted 1:10000 (Sigma). A chemiluminescence signal was detected by luminol substrate reaction (ECL Western Blotting System, GEHealthcare®). Immunoblots were quantified by membrane scanning in an Image4000, GE Healthcare®, and optical densities of proteins studied were determined by ImageJ software (Packard Instrument Company) and the protein/actin ratio calculated.

### Protein measurement

Protein was measured by Lowry’s method, modified by Peterson, using bovine serum albumin as a standard [54].

### Statistical analysis

All results were expressed as mean ± standard error mean (SEM) and analyzed by one-way analysis of variance (ANOVA) followed by Tukey’s or Dunnett’s test. The level of statistical significance was set at *p* < 0.05. All analyses were performed using the Prism 5.0 software (GraphPad).

## Results

### S100B secretion in the acute hippocampal slices of epileptic rats

**Fig. 1:**
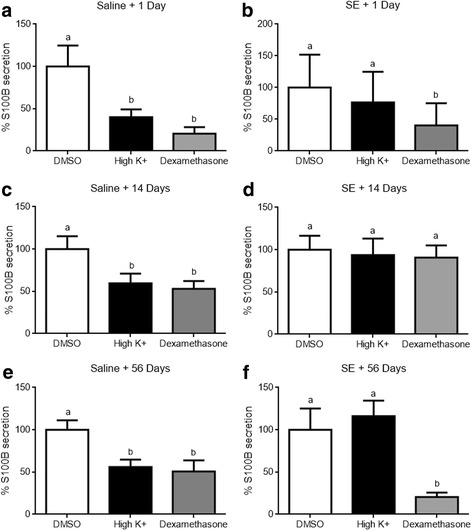
Dexamethasone and high potassium levels modulate S100B secretion in the acute hippocampal slices. S100B secretion from the hippocampus was measured by ELISA. Dexamethasone and high potassium levels decreased S100B secretion in sham (saline) animals at 1, 14, and 56 days (**a**, **c**, **e**). Dexamethasone reduced S100B secretion at 1 and 56 days after pilocarpine injection. SE animals were not affected by high potassium at 1, 14, and 56 days (**b**, **d**, **f**). Data are expressed as percentages compared to the basal condition and values represent mean ± standard error, of six to eight animals per group. Data were analyzed by ANOVA, followed by the Dunnet test. Bars without a common letter differ significantly, assuming *p* < 0.05

All animals from the Li-pilocarpine group, used in neurochemical assays, reached at least phase 4 of the convulsive Racine’s scale within 13 min after pilocarpine administration (data not shown). At 1, 14, and 56 days after Li-pilocarpine injection, we analyzed basal S100B secretion or S100B secretion in the presence of dexamethasone or in high-potassium medium in the acute hippocampal slices from sham and Li-pilocarpine-treated animals (Fig. 1).

S100B secretion in the hippocampal slices from sham animals is presented in panels a, c, and e, which correspond to 1, 14, and 56 days after saline administration. Li-pilocarpine-treated animals are shown in panels b, d, and f. Basal secretion did not differ between the sham and Li-pilocarpine-treated rats at all times (data not shown) and was assumed as 100%. Dexamethasone downregulated S100B secretion (*p* = 0.0073 in a, *p* = 0.0184 in c, and *p* = 0.0171 in e) in high-potassium medium at all times analyzed. Ex vivo S100B secretion of the hippocampi after SE induction of rats by Li-pilocarpine was not different in the normal- or high-potassium medium for all times analyzed. However, dexamethasone downregulated S100B secretion at 1 (panel b) and 56 days (panel f) following SE induction (*p* = 0.0184 and *p* = 0.0171, respectively). Dexamethasone did not affect S100B secretion at 14 days (panel c) after SE induction (*p* = 0.9242).

### In vivo dexamethasone prevents the increment in inflammatory cytokines, prostaglandin E2 and, cyclooxygenases

**Fig. 2:**
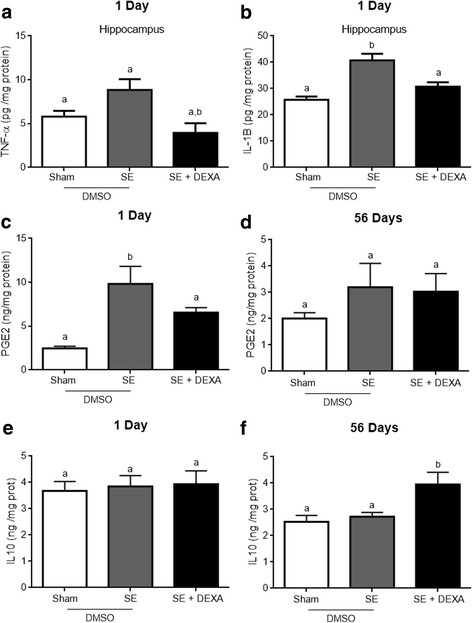
Dexamethasone prevents neuroinflammation in the hippocampus. Pro-inflammatory and anti-inflammatory cytokines were measured by ELISA. Dexamethasone decreased TNFα (**a**), reversed IL-1β (**b**), and PGE-2 (**c**) levels at 1 day after treatment. Dexamethasone did not affect PGE2 (**d**) content at 56 days nor IL-10 levels at 1 day after treatment. At 56 days, dexamethasone increased IL-10 content. Values were expressed by mean ± standard error, of four to six animals per group. Data were analyzed by ANOVA, followed by the Tukey test. Bars without a common letter differ significantly, assuming *p* < 0.05

Dexamethasone, administered at 24 and 36 h after SE induction, was able to prevent inflammatory signals of hippocampal inflammation caused by SE induction (Fig. 2). Dexamethasone prevented the augmentation in IL-1β (panel a) and PGE2 (panel c) levels in the hippocampus of SE animals at 1 day after pilocarpine injection (*p* = 0.0001 and *p* = 0.0002, respectively). The treatment with dexamethasone reduced TNF-α levels (panel b) when compared with SE and sham animals (*p* = 0.0286). No change was observed in IL-10 at 1 day after SE, and dexamethasone did not affect the levels of this anti-inflammatory cytokine at this time (*p* = 0.9221; panel d). However, interestingly, we observed an increase in hippocampal IL-10 in dexamethasone-treated animals at day 56 after SE (*p* = 0.0165, panel e). At 56 days after SE, the increment in PGE2 was not significant (*p* = 0.572; panel f) and dexamethasone did not affect this parameter.

**Fig. 3:**
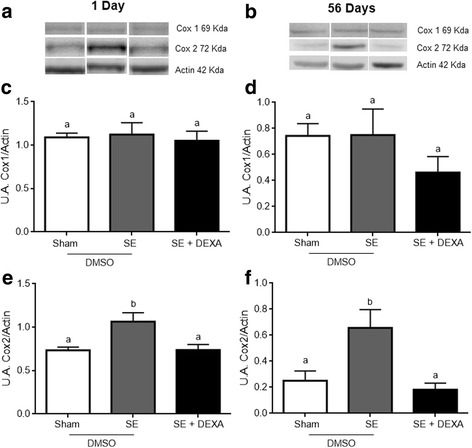
Dexamethasone prevents COX2 content in the hippocampus. COX1 and COX2 content were measured by Western blot. Representative images of COX1 and COX2 in the hippocampus at 1 and 56 days after treatment (**a**, **b**). Chemiluminescent quantification of protein/actin of COX 1 (**c**, **d**) and COX 2 (**e**, **f**). Dexamethasone prevented the increase in COX2 content in the hippocampus of SE animals at 1 and 56 days after treatment (**e** and **f**, respectively). Values represent mean ± standard error, of four to six animals per group. Data were analyzed by ANOVA, followed by the Tukey test. Bars without a common letter differ significantly, assuming *p* < 0.05

The cyclooxygenase (COX1 and 2) content was measured by Western blotting (Fig. 3). The immunocontent of COX1 was the same in all groups at 1 (panel c) and 56 (panel d) days (*p* = 0.8938 and *p* = 0.4244, respectively). However, dexamethasone prevented the increase in COX2 at 1 (panel e) and 56 (panel f) days (*p* = 0.0109 and *p* = 0.00913, respectively).

### Dexamethasone prevents astrogliosis markers, GFAP, and S100B in the epileptic model

**Fig. 4:**
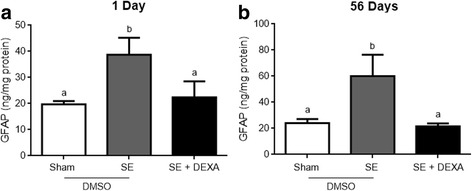
Dexamethasone prevents astrogliosis in the hippocampus. GFAP was measured by ELISA. Dexamethasone prevented the increase in GFAP content in the hippocampus of SE animals at 1 and 56 days after treatment (**a** and **b**, respectively). Values represent mean ± standard error, of four to six animals per group. Data were analyzed by ANOVA, followed by the Tukey test. Bars without a common letter differ significantly, assuming *p* < 0.05

**Fig. 5:**
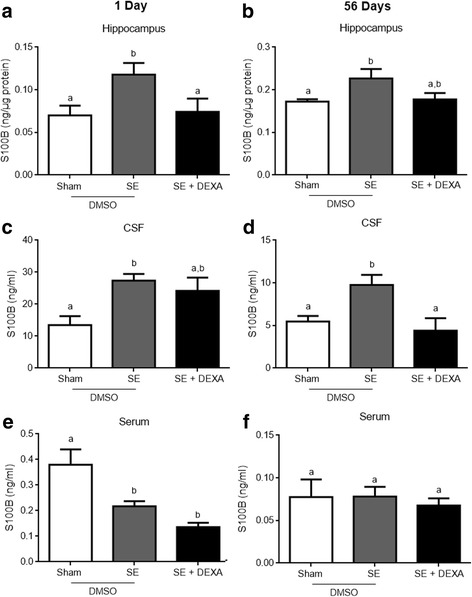
Dexamethasone modulates S100B levels in the hippocampus and in the cerebrospinal fluid. S100B content was measured by ELISA. Dexamethasone prevented the augmentation in S100B content in the hippocampus of SE animals at 1 day after treatment (**a**) and did not affect S100B content at 56 days after SE induction (**b**). The augmentation in S100B levels in the CSF was prevented by dexamethasone at 56 days after SE induction (**d**). Serum S100B levels were not altered by dexamethasone at any of the time points (**e**, **f**). Values represent mean ± standard error, of four to six animals per group. Data were analyzed by ANOVA, followed by the Tukey test. Bars without a common letter differ significantly, assuming *p* < 0.05

The increment in GFAP, induced in the Li-pilocarpine model of epilepsy, was prevented by dexamethasone in animals at 1 (Fig. 4a) and 56 (Fig. 4b) days after pilocarpine injection (*p* = 0.0035 and *p* = 0.0245, respectively). Hippocampal S100B content also increased following SE induction (Fig. 5a, b), and this was prevented by dexamethasone (panel c, *p* = 0.0062). However, the elevation in hippocampal S100B at 56 days in SE animals was partially prevented by dexamethasone administration (panel b, *p* = 0.0445).

### Dexamethasone prevented the increment in cerebrospinal fluid S100B during the chronic phase of the epileptic model

CSF S100B content was increased after SE induction at 1 (panel c) and 56 (panel d) days (*p* = 0.0125 and *p* = 0.0096, respectively). Dexamethasone was unable to prevent this increase at 1 day after pilocarpine administration (panel c). However, it completely prevented the elevation in CSF S100B at 56 days (panel c). Serum S100B content diminished in SE animals at 1 day (*p* = 0.0005; panel e), and dexamethasone did not affect this change. At 56 days, serum S100B was not different in SE animals or affected by dexamethasone (*p* = 0.3719; panel e).

### Dexamethasone prevents the decrease in glutathione in the Li-pilocarpine model of epilepsy

**Fig. 6:**
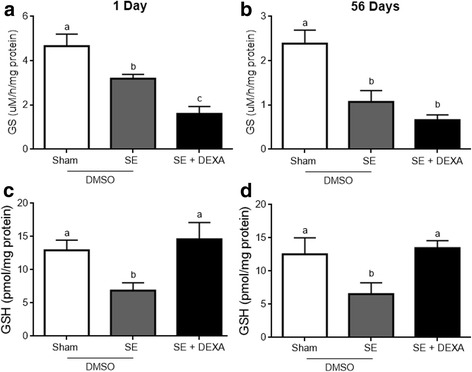
Dexamethasone does not modulate decreased GS activity but reverses GSH content in the hippocampus. The decrease in GS activity was not modulated by dexamethasone in the SE animals (**a**, **b**). Dexamethasone prevented the reduction in GSH reduced at 1 and 56 days after treatment (**c**, **d**). Values represent mean ± standard error, of four to six animals per group. Data were analyzed by ANOVA, followed by Tukey test. Bars without a common letter differ significantly, assuming *p* < 0.05

Based on the astrogliosis signals found in this model, we investigated other astroglial parameters related to astrocyte functionality, namely, glutamine synthetase (GS) activity, GSH content, potassium channel Kir 4.1, and aquaporin-4 (AQP-4). GS activity decreased in epileptic animals (Fig. 6) at 1 (panel a) and 56 (panel b) days (*p* = 0.0003 and *p* = 0.0030, respectively), and dexamethasone did not prevent this change at 1 or 56 days. However, we found a decrease in the hippocampal GSH content at 1 (panel c) and 56 (panel d) days (*p* = 0.0174 and *p* = 0.0500, respectively), possibly reflecting astroglial dysfunction, and dexamethasone administration completely prevented this alteration. In fact, GSH content is not an appropriate marker for astrocytes, but in the brain tissue, its synthesis and recycling are totally dependent on astrocyte activity.

### Dexamethasone prevents the impairment in potassium uptake by astrocytes in epileptic rats

**Fig. 7:**
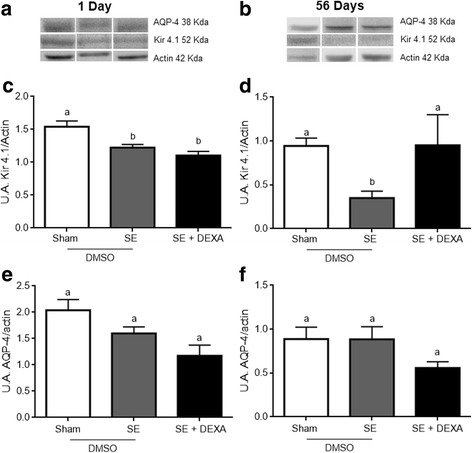
Dexamethasone does not alter Kir 4.1 and AQP-4 astrocyte channel content in the hippocampus. Kir 4.1 and AQP-4 contents were determined by Western blot. Representative images of Kir 4.1 and AQP-4 in the hippocampus at 1 and 56 days after treatment (**a**, **b**). Chemiluminescent quantification of Kir 4.1 (**c**, **d**) and AQP-4 (**e**, **f**) protein/actin. Dexamethasone did not reverse the reduction in Kir 4.1 content in the hippocampus of SE animals at 1 day after treatment (**c**); however, dexamethasone prevented Kir4.1 content in SE animals at 56 days after treatment (**d**). No differences in AQP-4 channel expression were observed between groups (**e**, **f**). Values represent mean ± standard error, of four to six animals per group. Data were analyzed by ANOVA, followed by Tukey test. Bars without a common letter differ significantly, assuming *p* < 0.05

Levels of the Kir 4.1 protein, the main potassium channel in astrocytes, was lower in epileptic rats at 1(Fig. 7c) and 56 (Fig. 7d) days (*p* = 0.0024 and *p* = 0.0418, respectively), and dexamethasone was able to prevent this effect at 56 days after SE induction, but not at 1 day after. There were no differences in AQP-4 levels in epileptic animals at 1 (Fig. 7e) and 56 (Fig. 7f) days (*p* = 0.6905 and *p* = 0.1419, respectively), and dexamethasone did not affect AQP-4 content at 1 and 56 days after SE induction.

## Discussion

Most AED target neuronal activity modulated by ionic channels, particularly GABA_A_. The steroid sensitivity of these channels has led to the use of steroids as adjunctive drugs for epilepsy [55–57]. Moreover, 30% of TLE patients develop resistance to AED [58], and additional strategies for therapeutic approaches are welcome. Mounting evidence suggests that astrocytes and neuroinflammation (which is modulated by astrocytes and microglia in brain tissue) contribute to epileptogenesis and are potential targets for therapies being developed against epileptic disorders [59].

### Dexamethasone affects S100B secretion

As previously mentioned, S100B is widely used as a marker for epileptic disorders [31, 32], and its secretion is modulated by anti-inflammatory drugs [35, 36]. Our studies have suggested that the hippocampal and CSF S100B are altered after SE induction in the Li-pilocarpine model [14]. We, herein, confirm that the hippocampal slices incubated in high-potassium medium secrete less S100B [60, 61]; this effect was observed in slices from young (14 days old) and adult (70 days old) sham rats. The mechanism underlying this decrease in S100B secretion remains unclear and could be mediated by an undetermined neuronal factor released during high-potassium depolarization, such as glutamate [60]. On the other hand, a direct effect of high-potassium on potassium channels and transporters in astrocytes modulating S100B secretion cannot be ruled out [61].

However, in the hippocampal slices from rats submitted to the Li-pilocarpine model of epilepsy, S100B secretion did not change in high-potassium medium, although the reason for this lack of change in S100B secretion is unclear at the moment [60], as is the mechanism by which S100B secretion occurs [37]. However, it is possible that the decrease in potassium channels in SE animals may contribute to the lower potassium influx in the astrocytes of these animals [49, 62, 63].

A number modulators of S100B secretions have been described [38]. Dexamethasone per se decreased S100B secretion in the acute hippocampal slices from sham animals (at all analyzed times) and in SE animals (at 1 and 56 days after pilocarpine administration). No in vitro effect of dexamethasone occurred at 14 days after SE, which corresponds to the “silent period” of this model [44]. Interestingly, these time-dependent changes in S100B secretion of the hippocampal slices of SE animals coincide with changes of this protein observed in CSF in the Li-pilocarpine model of epilepsy [14]. The acute effect of dexamethasone on the epileptiform activity of the hippocampal slices has been previously reported [64], but the mechanism underlying this activity remains unclear. Based on the effect of dexamethasone on S100B secretion at 1 day after SE induction, we decided to investigate whether dexamethasone administration at 24 and 36 h after pilocarpine injection prevents changes in inflammatory and astroglial markers at 1 and 56 days after dexamethasone injection.

### In vivo dexamethasone prevents neuroinflammation

The long-term effect of dexamethasone observed in astroglial and inflammatory parameters involves changes in gene expression, whereby dexamethasone prevents the increase in IL-1β, TNF-α, and COX2 (and consequently PGE2 levels) in the hippocampus of SE animals at 1 day after dexamethasone administration. Of note, in SE animals treated with dexamethasone, TNF-α was decreased to levels that were lower than those of sham animals, but unfortunately, we did not carry out cytokine measurements in the sham group without dexamethasone. Furthermore, dexamethasone increased IL-10, an anti-inflammatory cytokine, in SE animals at 56 days. Taken together, these data suggest the induction of a non-inflammatory microenvironment by dexamethasone in the hippocampus of Li-pilocarpine-treated animals over the short and long term.

### Dexamethasone prevents astrogliosis

Based on two classical glial markers, GFAP and S100B, we found that dexamethasone administration at 24 and 36 h after pilocarpine-induced SE administration was able to prevent astrogliosis. It is well known that GFAP and S100B expressions are downregulated in glial cultures by dexamethasone [36, 65] and that the in vivo administration of this corticoid has been used to reduce the inflammatory response and gliosis [66, 67]. Moreover, although dexamethasone was not able to reduce CSF S100B during a short time (2 days after SE), it was effective later on (at 56 days after SE). This effect may be of relevance as chronically elevated levels of this protein contribute to neurodegenerative diseases [37, 38]. Notably, we found a decrease in serum S100B after SE. This could be due to brain “retention” of this protein, as proposed in some cases of acute brain injury [68] or could be due to its peripheral alteration (independent of brain source) [69]. It is also important to mention that, in another model of SE induction using scopolamine/pilocarpine, an increase in serum S100B was reported and that the previous administration of dexamethasone prevented this increment [70].

### Dexamethasone protects against astrocyte dysfunction

Glutamine synthetase (GS) is a specific astrocyte enzyme that is critical to glutamate metabolism in the brain and closely related to glutamatergic and gabaergic neurotransmission and reduced in the human hippocampus in TLE [71]. A reduced expression of GS was reported at 2 weeks after SE in the Li-pilocarpine model [72]. In this study, we detected an earlier decrease in GS that persisted until the chronic phase. It is well known that dexamethasone induces the expression of this enzyme [73]; however, inflammatory cytokines are able to block this induction in astrocyte cultures [74]. We assumed that the hippocampal GS decrease in the Li-pilocarpine model is due to inflammation, but it is unclear at the moment why dexamethasone did not prevent this effect. It is possible that this effect may depend on the dose and time of corticoid administration. On the other hand, the hippocampal oxidative stress observed in this model [75, 76], characterized here by the decrease in GSH, was completely reversed by dexamethasone administration. Rosiglitazone, an agonist of the peroxisome proliferator-activated receptor gamma that has anti-inflammatory activity, also prevented the GSH imbalance in the hippocampus after pilocarpine-induced SE [77].

Neuronal excitability is highly dependent on extracellular levels of K^+^, which are regulated mainly by the astrocytic Kir 4.1 potassium channels that are in turn functionally coupled to the AQP-4 water channels [30]. In a previous study reporting on the induction of SE in rats with pilocarpine (without lithium), an increase in Kir 4.1 in the cortical regions, but not in the hippocampus, was observed at 8 weeks after SE induction [78]. We found an early (2 days after SE) and persistent (56 days after SE) decrease in Kir 4.1 content in the hippocampus. This apparently contradictory result is possibly due to methodological differences. Dexamethasone prevented the decrease in Kir 4.1 at 56 days after SE induction, but not at 1 day afterward. Accordingly, in the eye retina, dexamethasone (used to treat macular edema) selectively upregulated Kir 4.1 (but not AQP-4) channels [79]. Regardless of the changes in Kir 4.1 observed in SE animals, no changes in AQP-4 were observed in this model.

The use of corticosteroid therapy for epilepsy disorders is a matter of debate, due to the pro- and anticonvulsive effects observed. Experimental studies have administered dexamethasone before SE induction and observed behavioral alterations, changes in the latency period of SE, and mortality, biochemical, neurological, and inflammatory modifications [42, 80, 81]. However, if dexamethasone administration occurred during SE, there were no changes in latency period, increased mortality ratio, and exacerbated cerebral edema [43]. Data relating to COX2 expression in the experimental models suggests that the corticoid effect depends on the dose, the time point of administration, the type of inhibitor (selective or non-selective), and differences among models of SE induction [82].

**Fig. 8:**
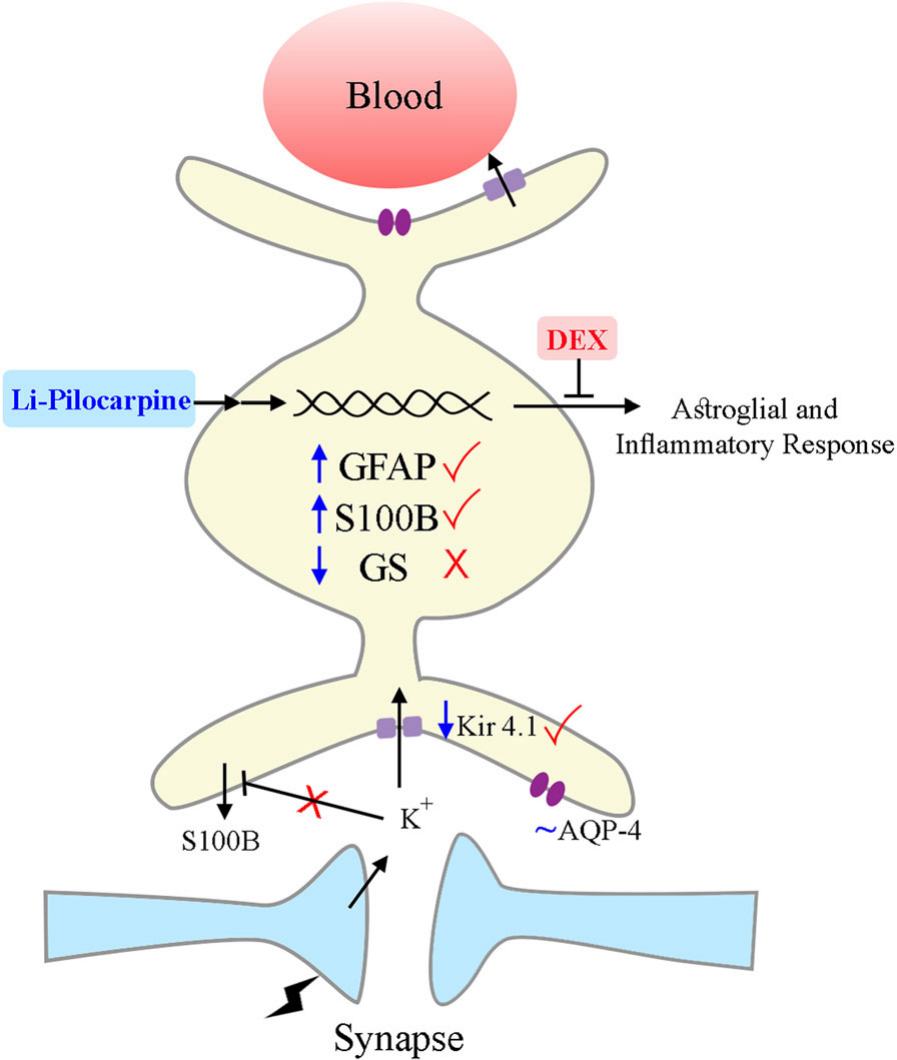
Schematic representation of astroglial alterations induced by Li-pilocarpine (at 56 days) and prevented by dexamethasone. We postulate that the Li-pilocarpine insult causes an inflammatory and astroglial injury. Dexamethasone (administered 24 h after pilocarpine) was able to prevent such alterations partially. Neuronal activity released K^+^, which is buffered by astroglial activity. GFAP, S100B, and GS expressions are sensitive to dexamethasone. The reduction in S100B secretion in the high-K^+^ medium was not observed in the hippocampal slices from Li-pilocarpine-treated animals. ~, ↑, and ↓ signify unchanged, increased, and decreased, respectively, caused by Li-pilocarpine administration; √ and X refer to the prevention or non-prevention, respectively, by dexamethasone (DEX)

Although dexamethasone clearly prevented astroglial and inflammatory changes, commonly associated to epileptogenesis, it did not alter the epileptic behavior, such as the beginning of recurrent spontaneous seizures and the scores on Racine’s scale (Additional file 2). We are assuming that dexamethasone effect involves a mechanism (direct or indirect) that alters the expression of these glial proteins, as it does with inflammatory proteins (cytokines and COX2). In fact, GFAP, GS, and S100B gene expression are sensitive to dexamethasone and cytokines [36, 83]. GS and GFAP exhibit differing sensitivities to dexamethasone in the hippocampus [36]. Herein, we observed that these proteins are differentially affected in the epilepsy model and that dexamethasone administration did not prevent the change in GS. Kir 4.1 and AQP-4 work together in the brain tissue to provide K^+^ and water clearance [30]. However, these channels were not affected in the same manner in this model of epilepsy—indicating impairment in the ability to remove K^+^. Moreover, to our knowledge, there are no data in the literature regarding the effect of dexamethasone on the gene expression of Kir 4.1; the administration of this corticosteroid prevented the decrease of these channels in the Li-pilocarpine model of epilepsy. Figure 8 summarizes the possible changes that occur in astroglial proteins in the model of epilepsy at 56 days after pilocarpine administration, as well as the prevention of such changes by dexamethasone administration. Notice that elevated extracellular levels of K^+^ decrease S100B secretion under basal conditions [60]. The mechanism involved in this effect is unclear at moment, but such an effect is not observed in the hippocampal slices from animals treated with pilocarpine. However, dexamethasone was able to reduce S100B secretion in the hippocampal slices from and epileptic rats in vitro. Moreover, our data allow us to speculate (but not affirm) that the protection provided by dexamethasone in this model could be useful against other epileptogenic agents such as traumatic brain injury or ischemia, in which neuroinflammation is present.

Some limitations of this study should be highlighted. Firstly, EEG records at different times would be useful to characterize SE and seizure activities later on. The absence of this data occurred in an effort to avoid surgery and the activation of neuroinflammation and astrogliosis by the lesion incurred by the introduction of electrodes. However, epileptic behavior analysis could provide valid evidence to suggest a protective role of dexamethasone in this model of epilepsy [84, 85]. Secondly, the different time points of 1, 14, and 56 days correspond to acute, latent (silent), and chronic epileptic phases, as determined in a previous study [49]. We did not perform a 14-day group in the second set of experiments to reduce the number of animals used in this study. Thirdly, this study demonstrates that dexamethasone administration (10 mg/kg, 24 and 36 h after SE) has clear neurochemical effects on the acute and chronic phases of Li-pilocarpine model of epilepsy. However, other protocols would be useful to delimit the best “window” and dose for steroid protection in this epilepsy model. Finally, dexamethasone prevented astroglial and inflammatory changes but did not alter the analyzed epileptic behavior, at least, between 7 and 25 days after SE induction, and therefore we cannot rule out the possibility that observed glial alterations constitute an epiphenomenon.

## Conclusions

Our results indicate a decrease in neuroinflammation, astrogliosis, and astroglial dysfunction in the hippocampi of young rats submitted to the Li-pilocarpine model of epilepsy, at 1 and 56 days after intraperitoneal dexamethasone administration. In the acute hippocampal slices prepared at 1, 14, and 56 days after SE induction, basal S100B secretion and S100B secretion in high-K^+^ medium were not different at 1 and 56 days, in contrast to sham animals in which high-K^+^ medium induced a decrease in S100B secretion. The addition of dexamethasone to the incubation medium per se induced a decrease in S100B secretion in sham and epileptic rats (1 and 56 days after SE induction). In the second set of experiments, we evaluated the in vivo effect of dexamethasone on hippocampal astroglial parameters (1 day after pilocarpine) in the epileptic model, at 2 and 56 days after SE. In addition to the improvement in inflammatory status (based on cytokine and PGE2 levels), dexamethasone prevented astrogliosis (based on GFAP and S100B content) and partially diminished astroglial dysfunction (based on Kir 4.1 protein and GSH content). The decrease in GS was not abrogated by dexamethasone, and AQP-4 was not altered in this epileptic model. All these parameters, with the exception of AQP-4, were altered, emphasizing the importance of this model for understanding alterations and mechanisms of epileptic disorders. In vivo dexamethasone administration, 24 h after SE induction, prevented most of the parameters analyzed, reinforcing the importance of anti-inflammatory steroid therapy in the Li-pilocarpine model and possibly in other epileptic conditions where neuroinflammation is present. Our data demonstrate specific alterations in astrocytes in this model and clearly contribute to the understanding of the importance of these cells in the pathogenesis of epilepsy, as well as suggest potential therapeutic targets for AED.

## Additional files

Additional file 1: Figure studies. Schematic experimental design. Schematic experimental design of the two studies. The first set of experiments analyze S100B secretion in an ex vivo model of hippocampal slices of rats, from sham and SE animals at 1, 14, and 56 days after pilocarpine injection. Hippocampal slices were incubated in high-K^+^ medium and dexamethasone. The second set of experiments evaluates dexamethasone treatment at 24 and 36 h after SE induction, in vivo at 1 and 56 days after dexamethasone injection. (TIFF 29 kb)

Additional file 2: **Table S1.** Behavior resumed. Epilepsy behavioral evaluation of SE animals. Evaluation of the occurrence of spontaneous epileptic seizures in animals submitted to the epilepsy model by Li-pilocarpine administration. Dexamethasone did not prevent behavioral changes. *All animals developed a spontaneous epileptic seizure and jumping and running behavior. (TIFF 16 kb)

## Abbreviations

AQP-4: Aquaporin channel 4
COX1: Cyclooxygenase 1
COX2: Cyclooxygenase 2
CSF: Cerebrospinal fluid
GFAP: Glial fibrillary acid protein
GS: Glutamine synthetase
GSH: Reduced glutathione
IL-10: Interleukin-10
IL-1β: Interleukin-1β
Kir 4.1: Inwardly rectifying potassium channel 4.1
LDH: Lactate dehydrogenase
PGE2: Prostaglandin E2
SE: Status epilepticus
TNF-α: Tumor necrosis factor-α
