# Supplementary figures and images for: Effects of dexamethasone on the Li-pilocarpine model of epilepsy: protection against hippocampal inflammation and astrogliosis
Source: J Neuroinflammation. 2018 Mar 5;15:68. doi: 10.1186/s12974-018-1109-5 (PMC5839012; doi:10.1186/s12974-018-1109-5)

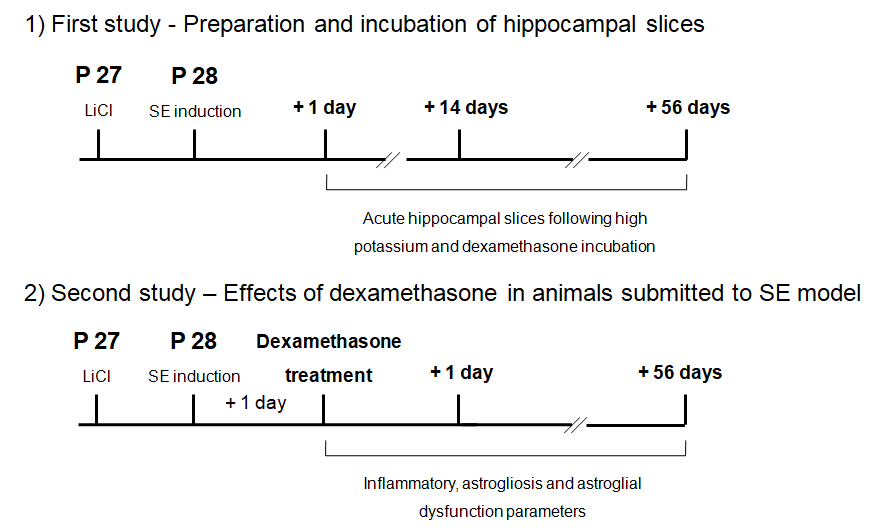

Supplement: Supplementary file 1 — Figure studies. Schematic experimental design. Schematic experimental design of the two studies. The first set of experiments analyze S100B secretion in an ex vivo model of hippocampal slices of rats, from sham and SE animals at 1, 14, and 56 days after pilocarpine injection. Hippocampal slices were incubated in high-K+ medium and dexamethasone. The second set of experiments evaluates dexamethasone treatment at 24 and 36 h after SE induction, in vivo at 1 and 56 days after dexamethasone injection. (TIFF 29 kb) [file 12974_2018_1109_MOESM1_ESM.tif]

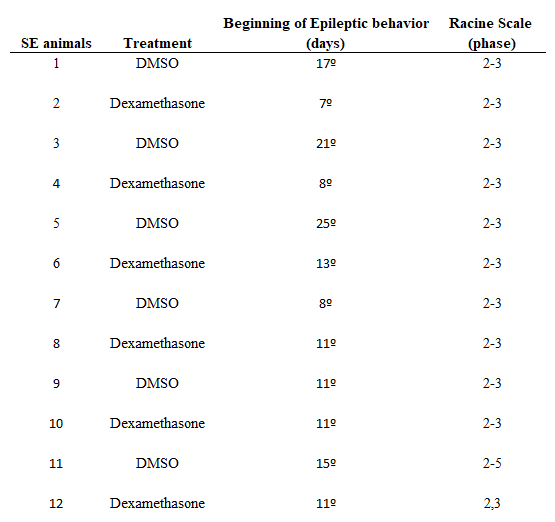

Supplement: Supplementary file 2 — Table S1. Behavior resumed. Epilepsy behavioral evaluation of SE animals. Evaluation of the occurrence of spontaneous epileptic seizures in animals submitted to the epilepsy model by Li-pilocarpine administration. Dexamethasone did not prevent behavioral changes. *All animals developed a spontaneous epileptic seizure and jumping and running behavior. (TIFF 16 kb) [file 12974_2018_1109_MOESM2_ESM.tif]
